# Effect of traditional Chinese medicine Bailing capsule on renal anemia in maintenance hemodialysis patients: A protocol for systematic review and meta-analysis

**DOI:** 10.1097/MD.0000000000029086

**Published:** 2022-04-15

**Authors:** Yan-Lin Li, Fang Cheng, Yan Chen, Jun Wang, Zeng-Dong Xiao, Bin Li

**Affiliations:** a Department of Pharmacy, Haibin People's Hospital of Binhai New Area, Binhai New Area, Tianjin, China; b Department of Traditional Chinese Medicine, Haibin People's Hospital of Binhai New Area, Binhai New Area, Tianjin, China; c Department of Psychiatry, Haibin People's Hospital of Binhai New Area, Binhai New Area, Tianjin, China.

**Keywords:** Bailing capsule, end-stage renal disease, maintenance hemodialysis, meta-analysis, renal anemia, traditional Chinese medicine

## Abstract

**Background:**

Renal anemia (RA) is one of the most common complications in patients with end-stage renal disease, and it is also one of the reasons for the decline of quality of life and functional status in patients with end-stage renal disease. Traditional treatment methods often fail to achieve satisfactory therapeutic effects, so it is very necessary to find effective adjuvant treatment methods. Bailing capsule (BLC), a traditional Chinese medicine, which has been widely used in the treatment of RA in maintenance hemodialysis patients, but a systematic review of the efficacy and safety of this drug is currently lacking. Therefore, this study used meta-analysis to evaluate the efficacy and safety of BLC in the treatment of RA, in order to provide guidance for finding effective auxiliary methods for the treatment of RA in maintenance hemodialysis patients (MHP).

**Methods:**

Using the computer to retrieve PubMed, EMbase, Cochrane Library, CNKI, VIP Database, WANFANG Database, SinoMed from 1990 to 2021 and collecting the clinical randomized controlled trial and retrospective cohort study of BLC in the treatment of RA in MHP. Two researchers independently read and screened the literature, followed by evaluating the retrospective cohort studies that met the selection criteria using the Newcastle-Ottawa Scale (NOS) scale. The randomized controlled trial used the Cochrane manual standards to assess the risk of bias, and the RevMan 5.3 software was used to conduct a meta-analysis of the result data.

**Results:**

This study will use the method of meta-analysis to evaluate the clinical efficacy and incidence of adverse reactions of BLC in the treatment of RA in MHP through the primary and secondary outcome indicators.

**Conclusion:**

The results of this study will help clinicians find safe and effective adjuvant therapy in the treatment of RA in MHP.

**OSF registration number:**

DOI 10.17605/OSF.IO/732KP (https://osf.io/732kp).

## 1 Introduction

End stage renal disease (ESRD), as the terminal stage of the development of various renal diseases under the condition of ineffective treatment, has gradually evolved into a global public health problem that seriously endangers human health. The number of patients accounts for about 8% ∼ 16% of the global population.^[1]^ Because the glomerular filtration rate of ESRD patients is significantly reduced, the metabolic waste, water, electrolytes and other substances excreted by the kidneys are reduced, and they accumulate in the body, resulting in a series of clinical manifestations and complications.^[2,3]^ Among them, renal anemia has become one of the most common complications of ESRD patients, mainly due to the reduction of erythropoietin (EPO) produced by the interstitial cells in the renal tubules. Renal anemia (RA) poses a variety of hazards to patients, including its ability to reduce the patient's quality of life, to increase the risk of cardiovascular events, and to be directly related to the development and progression of renal disease.^[4,5]^ Traditional clinical medicines for renal anemia include erythropoietin receptor activator (ERA), iron, folic acid, vitamin B_12_, etc.^[6]^ ERA is widely used in the treatment of patients with renal anemia and plays a crucial role in the clinical treatment of renal anemia, but the dose of ERA required to maintain target hemoglobin levels varies widely among patient populations. Even if ERA is used reasonably, there are still about 5% to 10% of patients with ESRD who are clinically hyporesponsive to ERA. In order to make the hematocrit and hemoglobin content of these patients reach the target level, the dose of ERA needs to be increased. This increases the chance of adverse drug effects, including an increased risk of cardiovascular events and death.^[7]^ Therefore, it has been recognized that in addition to the traditional treatment of RA, appropriate adjuvant therapy may help improve the anemia status of patients.

Doctors in China have focused on seeking drugs with significant curative effect and relatively few side effects in the field of traditional Chinese medicine (TCM) to treat RA, and have made some progress. Among them, the Bailing capsule (BLC) has been widely used in the treatment of RA in maintenance hemodialysis patients (MHP). Traditional Chinese medicine BLC is made by low-temperature fermentation of *Cordyceps sinensis*. Its main components are Cordyceps oxalic acid, D-Mannitol, 19 amino acids, carrier alkaloids, a variety of trace elements and vitamins. At present, it has been widely used in the treatment of ESRD and its complications.^[8]^ Due to the lack of a systematic review of the efficacy and safety of BLC for RA in MHP. Therefore, this study evaluated the efficacy and safety of BLC in the treatment of RA by means of meta-analysis, in order to provide research ideas for finding an effective auxiliary method for the treatment of RA in MHP.

## 2 Methods

### 2.1 Study registration

This study has been registered on the OSF. (Registration number: DOI 10.17605/OSF.IO/732KP, registered website: https://osf.io/732kp).

### 2.2 Eligibility criteria

#### 2.2.1 Study types

To collect literatures related to BLC in the treatment of RA in MHP. The types of literature were randomized controlled study and retrospective study. Whether or not the random assignment method is described. The language of literature retrieval is set as Chinese or English.

#### 2.2.2 Participants

The enrolled population was patients with confirmed renal anemia on maintenance hemodialysis, and their general information such as age, gender, race, scope of disease and education level are not restricted.

#### 2.2.3 Inclusion criteria

- Those who meet the diagnostic criteria of Western medicine and TCM.
- The age range is 18 to 75 years old, male or female.
- Those who have not taken *C* sinensis preparations within 3 months.
- Regular maintenance of hemodialysis for more than 6 months, 8 to 12 hours of dialysis per week, and stable condition.

#### 2.2.4 Exclusion criteria

- Anemia caused by other causes, such as blood loss anemia, hemolytic anemia, and aplastic anemia.
- Patients with other serious infections, severe heart failure and systemic diseases.
- The types of studies are reviews, conference papers, meta-analyses, and animal experiments.

### 2.3 Interventions

The control group was given only drugs to stimulate erythropoiesis. The experimental group was given BLC, and all patients could be given the corresponding basic treatment according to the patient's condition.

### 2.4 Types of outcomes

#### 2.4.1 Primary outcomes

Hemoglobin content, hematocrit, ferritin content, transferrin saturation.

#### 2.4.2 Additional outcomes

Serum albumin, serum creatinine, blood urea nitrogen, serum uric acid, endogenous creatinine clearance, C-reactive protein, and incidence of adverse reactions.

### 2.5 Search strategy

Using the computer to retrieve PubMed, EMbase, Cochrane Library, CNKI, VIP Database, WANFANG Database, SinoMed from 1990 to 2021 and finding out the clinical randomized controlled trials and retrospective cohort studies on the treatment of RA in MHP by BLC. The retrieval is carried out in the form of mesh terms combined with free words, and the retrieval terms are connected with “OR” or “AND”.

### 2.6 Research collection and data analysis

**Figure 1:**
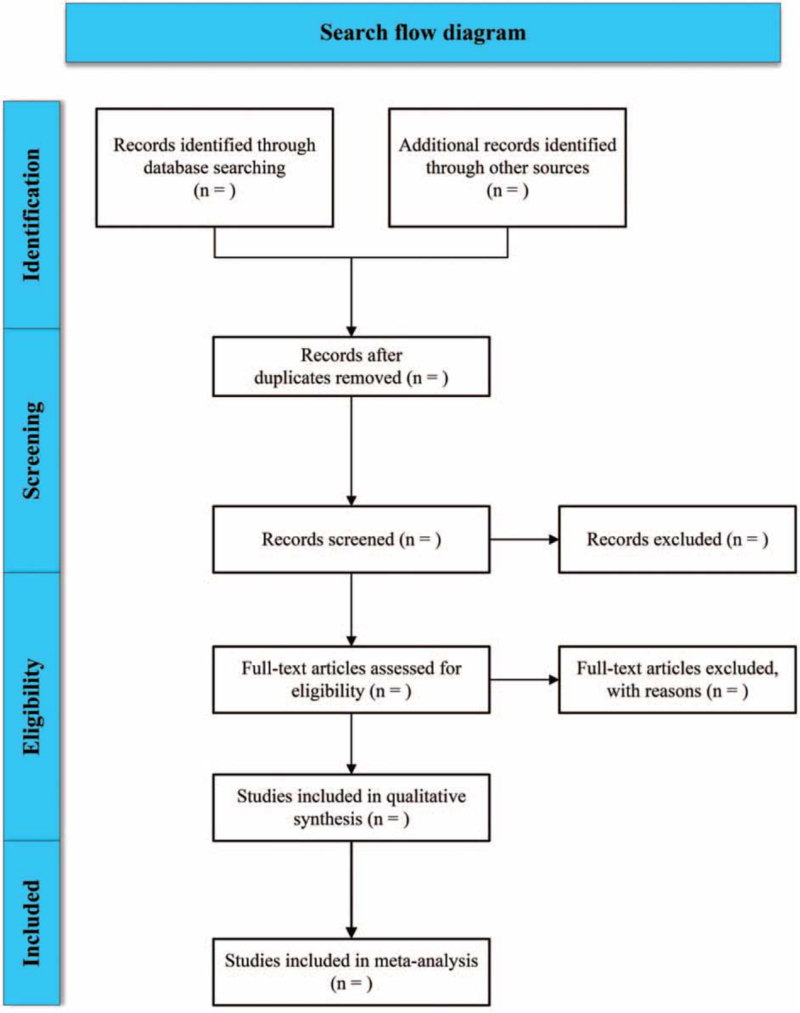
Flowchart of the process of searching complete relevant literature.

Two researchers independently screened the literature, extracted the data and checked each other in strict accordance with the screening criteria. If there is disagreement, discuss with the third researcher for decision. The extracted contents are: Basic information of literature: author, year, title, literature source, etc; Elements of bias risk assessment: random method, blind method, etc; Intervention measures: drug name, intervention duration, etc; Outcome indicators. The process of collecting and screening literature is shown in Figure 1.

### 2.7 Assessment of risk of bias

The bias risks included in the study are independently assessed by 2 researchers and cross-check separately. The included RCT used an independent evaluation of the Jadad score to include the bias risks in the study, with 3 and above 3 indicating higher quality. The retrospective cohort study experiment is evaluated using the NOS scale, with a score of 9, and the higher the score, the higher the quality of the literature, and the literature with more than 5 are of high quality.

### 2.8 Data synthesis and analysis

The data extracted from the literature are meta analyzed by RevMan 5.3 software (Cochrane, UK). Firstly, the heterogeneity among the included literatures is analyzed by *I*^2^ test. When the heterogeneity among the literatures is small (*I*^*2*^≤50%, *P* > .1), the fixed effect model is used for analysis; When the heterogeneity among literatures is large (*I*^2^ > 50%, *P* < .1), the random effect model is used for analysis, and the obvious clinical heterogeneity is treated by subgroup analysis or sensitivity analysis. The difference in mean difference is used as the effect index for the continuity index data, and the relative risk or odds ratio is used as the effect index for the 2 classification index data. The 95% confidence interval is calculated respectively, and *P* < .05 is the difference, which has significant significance.

### 2.9 Sensitivity analysis

For those who have heterogeneity between the research data, one by one, to exclude the data of a single study, to see if the results have changed substantially. If the results of a single study have changed significantly, then the literature may be the source of heterogeneity.^[9]^

### 2.10 Subgroup analysis

Subgroup analysis will be performed on the results of studies with greater heterogeneity. The subgroup analysis will be based on the integrity of the available data in the literature in terms of patient age, course of illness, whether or not to combine medication, total duration of treatment, geography, and race.

### 2.11 Publication bias

The funnel plot is drawn using RevMan 5.3 software to determine the size of the publication bias by observing the symmetry of the funnel plot. The more symmetrical the funnel is, the smaller the publication bias is, and vice versa.

### 2.12 Evidence evaluation

GRADE system is a set of evidence evaluation system, which is widely used in systematic evaluation, health technology evaluation and guide production because it is more representative than other evidence evaluation systems. GRADE classes the evidence system into 4 levels: high, medium, low, and very low. The quality shows whether further studies will affect or alter the evaluation result of the effectiveness. In this study, the GRADE (Pro 3.6) software (The Grading of Recommendations Assessment, Development and Evaluation Working Group) is used to evaluate the evidence quality in the RCT study of the result variables in this study.

### 2.13 Ethics and dissemination

The systematic review does not involve new clinical trials. All data are extracted from published literature, so ethical approval and patient informed consent are not required.

## 3 Discussion

Anemia is one of the most common complications of ESRD patients during maintenance hemodialysis, and it is also one of the reasons for the decline of quality of life and body function of ESRD patients. Correcting anemia is a vital goal in the management of RA patients with maintenance hemodialysis.^[10,11]^ BLC is a low-temperature fermentation of *C sinensis* strains. Studies have shown that the *Cordyceps mycelium* contained in BLC can promote the synthesis of protein and amino acids in the body, enhance the body's positive nitrogen balance, and also strengthen the hematopoietic function of bone marrow and promote the release of red blood cells. And prolong the life span of red blood cells, thereby improving the symptoms of protein deficiency and anemia in patients, which is conducive to the improvement of nutritional status.^[12]^ Although BLC has been used by clinicians in the treatment of RA in MHP, its efficacy and safety are still controversial. Due to the lack of relevant systematic review, this study is very valuable. This study will use the existing literature research results to actively guide clinical practice, promote the transformation of theoretical evidence into clinical practice, and supplement and improve the treatment methods of RA in MHP from the perspective of TCM, Therefore, this study will play a positive role in improving the therapeutic effect of RA in MHP.

## Author contributions

**Conceptualization**: Bin Li, Yan-Lin Li, Fang Cheng.

**Data curation**: Yan-Lin Li, Fang Cheng.

**Formal analysis**: Yan-Lin Li, Fang Cheng, Yan Chen, Jun Wang.

**Funding acquisition:** Bin Li.

**Investigation**: Yan-Lin Li, Fang Cheng, Yan Chen, Jun Wang, Zeng-Dong Xiao.

**Methodology**: Yan-Lin Li, Fang Cheng.

**Resources:** Yan-Lin Li, Yan Chen, Zeng-Dong Xiao.

**Software:** Fang Cheng, Yan Chen, Jun Wang.

**Supervision**: Bin Li, Yan-Lin Li.

**Writing – original draft**: Yan-Lin Li, Fang Cheng, Yan Chen, Jun Wang, Zeng-Dong Xiao.

**Writing – review & editing:** Bin Li.
